# Adverse effects and perceived benefits of medications in children with chronic noncancer pain

**DOI:** 10.3389/fpain.2026.1794069

**Published:** 2026-04-20

**Authors:** Kacper Niburski, Nada Mohamed, Dominique Dundaru-Bandi, Victor Hugo Gonzalez Cardenas, Marie Vigouroux, Rebecca Pitt, Pablo Ingelmo

**Affiliations:** 1 Department of Anesthesiology, University of British Columbia, Vancouver, BC, Canada; 2 Edwards Family Interdisciplinary Center for Complex Pain, Montreal Children’s Hospital, McGill University Health Centre, Montreal, QC, Canada; 3 Faculty of Medicine and Health Sciences, McGill University, Montreal, QC, Canada; 4 Facultad de Medicina, Fundación Universitaria de Ciencias de la Salud - FUCS, Bogotá, Colombia; 5 Unitat de Dolor Pediàtric, Servei Anestesiologia i Reanimació, Hospital Sant Joan de Déu, Esplugues de Llobregat, Barcelona, Espanya; 6 Dipartimento di Medicina e Chirurgia, Universita Degli Studi Milano Bicocca, Milano, Italia; 7 SSD Terapia del Dolore e Medicina Palliativa Pediatrica, Fondazione IRCCS San Gerardo dei Tintori, Monza, Italia

**Keywords:** adverse effects, chronic pain, pharmacological treatments, primary pain conditions, secondary pain conditions, therapeutic benefits

## Abstract

**Background:**

There has been limited research in assessing the risk–benefit profile of pharmacological interventions for pediatric chronic pain.

**Aims:**

This quality-improvement analysis evaluated the incidence of adverse effects within the first month of initiating pharmacologic therapy in children and adolescents with chronic non-cancer pain receiving pharmacological treatment. In addition, we assessed perceived benefits of pharmacologic interventions during the same period.

**Methods:**

We included pediatric patients with chronic primary or secondary pain enrolled in tertiary care interdisciplinary pain program. The primary endpoint was the proportion of patients reporting adverse effects within four weeks of starting a new medication (antiepileptics, antidepressants, nonsteroidal anti-inflammatory drugs, Tramadol or other drugs). Patients were provided with a tabulated adverse effects (5% or higher) for each medication they were receiving. We quantified the incidence of adverse effects, but severity of the adverse effects, causality or pharmacological interactions were not assessed. The secondary outcome was the proportion of patients reporting benefit as a dichotomous variable (Yes or No) in pain, sleep, physical function and mood. Each domain was treated as an independent, non-composite variable without graded scales.

**Results:**

142 patients aged 7–17 with chronic, predominantly female (90%) received 291 prescriptions (mean 2.0 per patient). Adverse effects occurred in 115/142 patients (81%), totaling 572 events (mean 2.0 per patient). Gastrointestinal (62%) and attention-related (61%) were the most common reported adverse effects. Adverse effects increased with more concomitant medications (72% on one drug, 81% on two drugs, 95% on three or more). Perceived benefits were reported by 109 patients (77%), including analgesic benefit (64%), sleep benefits (49%), physical function (27%), and mood (22%). The mean number of benefited domains per patient increased with polypharmacy. However, the number of perceived benefits per prescription decreased with the number of medications prescribed.

**Conclusions:**

Adverse effects are common in the first four weeks of treatment, particularly with polypharmacy, alongside patient-perceived benefits in a real-world setting. These results are descriptive and do not establish causality, drug-specific efficacy, or interactions, but highlight the need for safety monitoring and engaged decision-making.

## 1 Introduction

Chronic pain, defined as persistent or recurrent pain lasting more than 3 months, is common in children and adolescents (1). About 20% of children report lifelong recurrent pain, with prevalence varying by pain type and higher rates in females (2). Chronic pain is linked to depression, anxiety, social isolation, school absences, and reduced quality of life. Approximately 5% experience high-impact chronic pain (3). These issues relate to lifelong comorbidities and functional disabilities in adulthood (4–6).

Interdisciplinary pain treatment programs are the gold standard for this vulnerable population, incorporating pharmacological treatments, physiotherapy, psychotherapy, and the expertise of pain physicians and nurses (7). Pharmacological interventions remain common and are often the only option when access to interdisciplinary care is limited (8). However, the data guiding pharmacological therapy for children and adolescents with chronic pain is limited and largely derived from adult studies, which raises concerns about efficacy and safety (8, 9). For example, gabapentinoids are often used as first-line treatment for pediatric neuropathic pain, despite no specific approval from the European Medicines Agency (EMA) or from the Food and Drug Administration (FDA). Antidepressants are commonly prescribed in pediatric pain management, mirroring adult protocols without specific approval from the FDA or EMA. Moreover, gabapentinoids and antidepressants are linked to an increased risk of suicidal ideation in young people, with EMA and FDA warnings issued for adolescent use (10).

There has been limited progress in assessing the risk–benefit profile of pharmacological interventions for pediatric chronic pain in recent years (8). The Pediatric Research Equity Act (PREA), implemented in 2003, gives the FDA the authority to require pediatric studies in certain drugs, biologics, and vaccines. However, the law also stipulates that pediatric effectiveness can be extrapolated from well-controlled studies in adults, if the course of disease and effect of the drug are sufficiently similar in children and adults. The Best Pharmaceuticals for Children Act (BPCA) provides incentives for voluntary pediatric studies and has been in effect since 2002, with reauthorizations in 2007, 2012, 2017, and 2022. In September 2010, the NIH established the Pediatric Trial Network (PTN) to facilitate pediatric clinical drug trials. Yet studies on chronic pain medications for children remain scarce (10).

Research on treatment efficacy typically assesses whether an intervention yields the intended clinical benefit under ideal conditions, using validated scales or endpoints. Such evaluations tend to be quantitative, disease-specific, and focused on a single intervention and predefined outcomes. However, evaluating efficacy across multiple interventions, diagnoses, or in polypharmacy within interdisciplinary programs is more complex in real-world settings (10).

Adverse effects (AEs) are defined as all unintended, undesirable effects that occur during drug use at standard doses, regardless of whether a causal link to the drug has been established. Adverse drug reactions (ADRs) are judged to be causally related to the drug, based on evidence from temporality, dechallenge/rechallenge, and pharmacovigilance assessment (11). Moreover, nocebo effects occur when negative expectations about a treatment increase adverse-event reporting or reduce perceived benefit, even without actual harm (12). There is a need for a better understanding of the potential risks and benefits associated with the medical treatment of pediatric chronic pain (8).

In pediatric interdisciplinary care, accounting for patient and caregiver expectations is essential to avoid confounded safety signals and to assess true treatment effects. Although multiple qualitative and quantitative frameworks exist to integrate benefit and risk into a composite metric for adults, these measures have not been applied to analgesic trials in children with chronic pain (10). In clinical practice, risks and benefits are addressed at prescription and assessed at follow-up visits. A quality improvement framework can test changes in real-world settings using the Plan, Do, Study, Act (PDSA) model. PDSA cycles enable rapid, iterative assessment and co-monitoring of adverse effects rates and perceived benefits to understand patient-level trade-offs for individual and combined treatments. This model could answer relevant questions like how frequent AEs are or if AEs are more frequent with polypharmacy. At the same time, we can evaluate the benefits perceived in the patient's perspective under real life clinical conditions (13, 14).

Therefore, the aim of this quality-improvement analysis was to evaluate the incidence of adverse effects within the first month of initiating pharmacologic therapy in children and adolescents with chronic non-cancer pain. In addition, we assessed perceived benefits of pharmacologic interventions in this population during the same period.

## 2 Methods

This quality improvement analysis evaluated the incidence of AEs and perceived benefits associated with medications prescribed at the Edwards Family Interdisciplinary Center for Complex Pain (CCP) of the Montreal Children's Hospital and was approved by the ethical committee of the McGill University Health Center (2019-467). The authors were granted permission not to collect consent due to the quality improvement aim of the project.

### 2.1 Patients

We included pediatric patients older than 6 years old with chronic primary or secondary pain enrolled in the interdisciplinary pain program. Included patients had either already been taking medications or were starting a new pharmacological treatment or both at the time of their initial evaluation with the clinical team. The sample included children or adolescence without cognitive disabilities able to describe adverse effects, their onset or potential benefits associated with the pharmacological treatments by themselves or with the assistance of their guardians. Patients with cancer pain, patients with cognitive disabilities or who could not report perceived benefits or adverse effects even with the assistance of their caregivers, or who did not wish to participate were not included in the study.

Patients evaluated by the clinical team were offered individualized physiotherapy, cognitive behavioral therapy (CBT), interventional treatments and pharmacological treatments (15). Patient-specific individual outpatient physiotherapy programs included desensitization, graded motor imagery, mirror therapy, stretching and postural changes. The CBT interventions were provided as group or individual outpatient sessions and included biofeedback and deep relaxation.

At our center, diagnoses were recorded using the IASP/ICD-11 framework, which generally provides adequate classification for organizing care. Given the multidimensional, personalized treatment program, emphasis is placed on domains such as pain, physical function, mood, and social functioning, with pharmacologic choices guided by pain and psychosocial phenotype rather than by a single diagnosis or pre-stablished protocols. Many patients referred to our tertiary center have overlapping symptoms or lack a specific diagnosis; the entry point and outcome focus is to reduce pain-related disability and help families return to daily life rather than pursue a disease-specific cure.

Pharmacological treatments were offered using a protocol based on the individual pain sensation within the CCP's multimodal pain treatment program (16, 17). These medical interventions included the use of antiepileptics, antidepressants and nonsteroidal anti-inflammatory drugs (NSAIDs). A short course oral Tramadol or a combination of Tramadol/Paracetamol for 2–4 weeks in acute on chronic situations, was offered to reduce pain intensity and facilitate physiotherapy rehabilitation in selected cases.

The efficacy of the treatment and the AEs are regularly documented by the team nurse within four weeks of starting medication during a scheduled phone call evaluation or after a spontaneous patient/parent report to the team nurse. There after we schedule follow-ups every 8–12 weeks (15).

### 2.2 Outcomes

The primary endpoint of the study was the proportion of patients with perceived AEs within four weeks of starting a new medication. Patients received a list of tabulated AEs (5% incidence or higher) as described in Lexicomp™, UpToDate™, and the manufacturer's information for each of the pharmacological treatments they were receiving or will receive. Less frequent AEs were acknowledged but not detailed in the list to balance safety with minimizing nocebo effects. The clinical team provided red-flag guidance and patients/parents were advised to report unusual symptoms and seek timely care as appropriate.

Three reviewers (NM, VG, PI) codified the language using a consensus-based approach to ensure consistency between adverse effects. The adverse effect profile sheets were coded in French and English, and in a manner similar to that used by Sandritter et al. (18). Patients were given one sheet for each medication they were taking either previously taking or new ones.

All participants enrolled in the program were required to have a parent or guardian present during the evaluations. Parents were invited to assist their children to complete the questionnaire independently or to contextualize the adverse effects and perceived benefit. Adverse effects and benefit questionnaires were completed by the patient alone when feasible. For children and adolescents unable to reliably report themselves due to age a parent/guardian provided proxy responses. In all cases, both the patient and the parent/guardian could participate if they wished.

**Figure 1:**
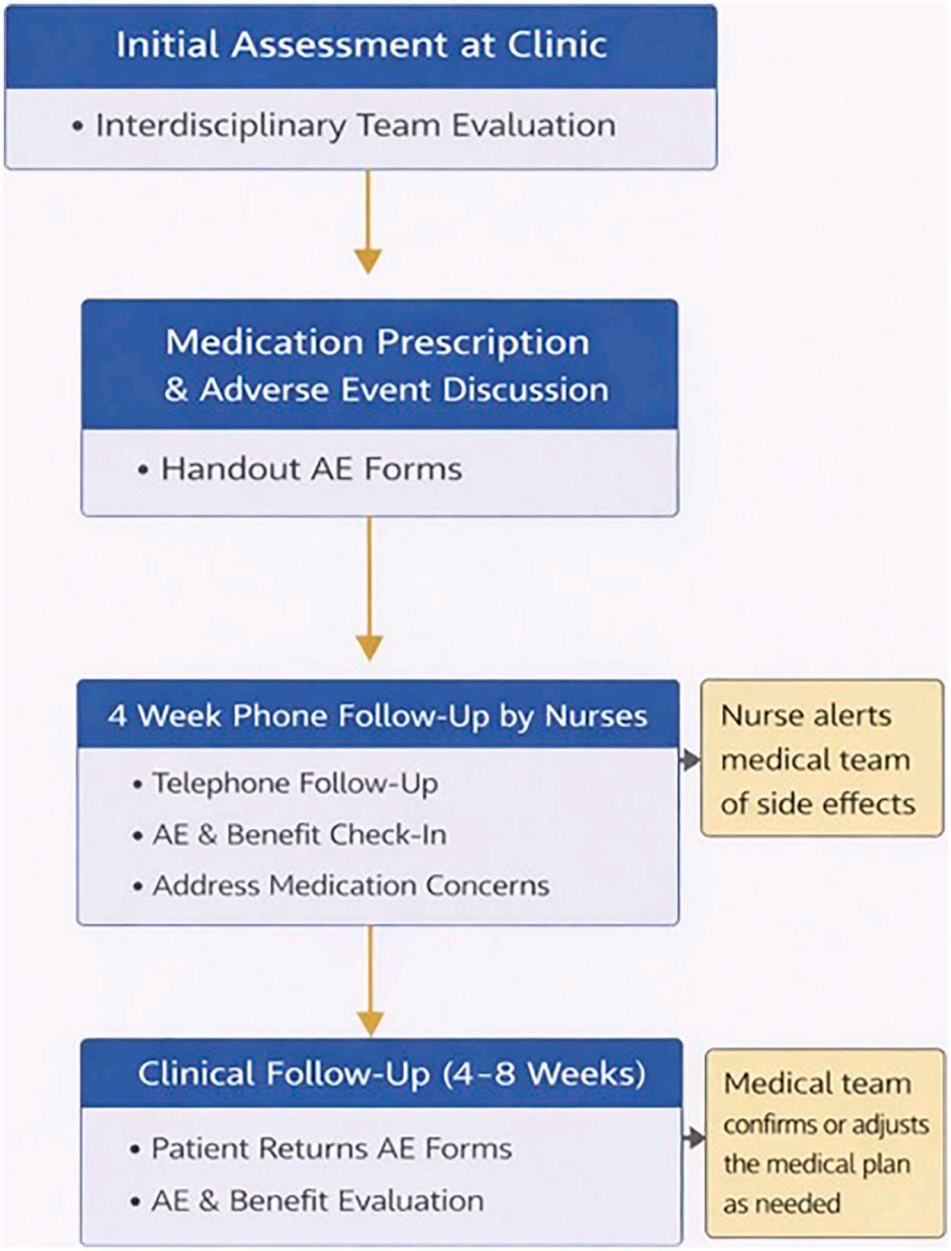
Flow chart showing the data collection process in the clinic.

Figure 1 describes the procedures and timing of the quality improvement evaluation. For the purposes of this quality improvement analysis and because most AEs are reported during the first weeks of treatment, we included the evaluations at four weeks. The aim of this quality improvement analysis was to estimate the incidence of AEs and to understand the implications of polypharmacy in our patient population at treatment initiation. The severity of the AEs was evaluated by the clinicians in charge of the clinical care but not reported in the quality improvement analysis. The clinical decisions (e.g., continuing or suspending treatment, dose modifications) related to the intensity of AEs were made by the treating team in consultation with the patients and their guardians.

The secondary outcome was the proportion of patients reporting perceived benefit across predefined domains, recorded during the first four weeks after initiating a new pharmacologic treatment. For each domain, participants (or their guardians when applicable) indicated whether they perceived a benefit (Yes) or not (No) in pain-related outcomes (pain intensity, pain duration, and pain frequency), sleep (sleep quality and sleep disruption due to pain), improve in physical function and positive mood changes. These items were treated as separate, non-composite variables. No graded scales or cumulative scores were constructed; each domain was analyzed as an independent indicator of perceived benefit.

A nurse clinician contacted the patients within four weeks of starting a new medication and collected the questionnaires (new and old medications) during the auditing period. Three reviewers (KN, NM, VG) independently screened and consolidated the data. The analysis of the adverse effects included the proportion of patients reporting AEs, the types of AEs, and the proportion of AEs associated to the number of medications received during the study period. The analysis of the perceived benefits included the proportion of patients reporting any perceived benefits in pain, sleep, mood or physical function and the proportion of patients reporting benefits with different number of simultaneous medications.

### 2.3 Statistics

Data were presented descriptively as either counts or percentages, and mean values were presented with standard deviation. This quality analysis was not powered or designed for formal causality or magnitude of the effect testing. Additionally, potential confounding factors and limited subgroup sizes would limit the reliability of any comparative inferences. Therefore, a formal statistical analysis including comparison between populations or interventions was not performed. Calculations were performed using Excel (Microsoft Office 2024).

## 3 Results

During 12 consecutive months, 142 of 150 patients between 7 and 17 years old, with chronic pain conditions received 291 prescriptions (mean 2 +/- 1 per patient) and completed the questionnaires. The study included predominantly female participants (approximately 90%), reflecting the sex distribution observed in our center's pediatric chronic-pain population. Eight patients were excluded from the study as they did not receive pharmacological interventions or refused participation in the quality improvement project.

Sixty-nine (49%) patients received one medication that was prescribed by the clinical team during the study period. Thirty-one patients (22%) received two medications, 42 patients (30%) received three or more medications. The medications most frequently prescribed were Celecoxib (46%), Gabapentin or pregabalin (28%,), Amitriptyline (22%), Melatonin (20%), Clonidine (18%), Tramadol (15%), Topiramate (6%) and Duloxetine (3%) (Table 1).

**Table 1:** Types of adverse effects (AEs) per patients, prescription and medications. Data are number and proportion (%) of patients reporting AEs, prescription related AEs and AEs associated with the use of medications for the treatment of chronic pain conditions. (1) Gastrointestinal AEs: abdominal pain/fullness, constipation/ diarrhea, decreased/increase appetite, nausea/vomiting, wight loss/gain, unpleasant taste, dry mouth. (2) AEs affecting attention: Decreased concentration, somnolence, dizziness, drowsiness, memory impairment, fatigue, sedation, blurred vision, involuntary eye movements. (3) AEs associated with uncoordinated movements and weakness: tremor, decreased coordination to fine movements, vertigo. (4) AEs affecting sleep: nightmares, insomnia. (5) AEs affecting the skin: itching, bruising, rush, sweating, swelling, cold. (6) Mood changes: irritability, depression, anxiety, hallucinations, hostility. (7) Increased pain: generalized pain, joint pain, muscular pain/spasms. (8) Other AEs: fever, cold/flu symptoms, urinary retention.

Adverse effects	Patients (n142)	Prescriptions (n291)	Medications	
Gastrointestinal (1)	88 (62%)	212 (73%)	Amitriptyline (n32)	6 (29%)
			Celecoxib (n66)	25 (38%)
			Clonidine (n26)	5 (19%)
			Gabapentin (n22)	9 (41%)
			Melatonin (n29)	4 (14%)
			Naproxen (n17)	3 (18%)
			Pregabalin (n24)	10 (42%)
			Tramadol (n21)	6 (29%)
Affecting attention (2)	87 (61%)	212 (73%)	Amitriptyline (n32)	9 (28%)
			Celecoxib (n66)	19 (29%)
			Clonidine (n26)	6 (23%)
			Gabapentin (n22)	6 (27%)
			Melatonin (n29)	11 (38%)
			Naproxen (n17)	5 (29%)
			Pregabalin (n24)	9 (38%)
			Tramadol (n21)	7 (33%)
Weakness/ Movements (3)	26 (18%)	29 (10%)	Amitriptyline (n32)	2 (6%)
			Celecoxib (n66)	6 (9%)
			Clonidine (n26)	3 (12%)
			Gabapentin (n22)	3 (14%)
			Melatonin (n29)	6 (21%)
			Naproxen (n17)	-
			Pregabalin (n24)	1 (4%)
			Tramadol (n21)	-
Affecting sleep (4)	25 (18%)	33 (11%)	Amitriptyline (n32)	3 (1%)
			Celecoxib (n66)	7 (10%)
			Clonidine (n26)	1 (4%)
			Gabapentin (n22)	5 (23%)
			Melatonin (n29)	2 (7%)
			Naproxen (n17)	2 (12%)
			Pregabalin (n24)	-
			Tramadol (n21)	1 (5%)
Affecting the skin (5)	23 (16%)	28 (10%)	Amitriptyline (n32)	2 (6%)
			Celecoxib (n66)	6 (9%)
			Clonidine (n26)	2 (8%)
			Gabapentin (n22)	3 (14%)
			Melatonin (n29)	-
			Naproxen (n17)	-
			Pregabalin (n24)	3 (13%)
			Tramadol (n 21)	3 (14%)
Mood changes (6)	21 (15%)	27 (9%)	Amitriptyline (n32)	-
			Celecoxib (n66)	5 (8%)
			Clonidine (n26)	2 (8%)
			Gabapentin (n22)	4 (18%)
			Melatonin (n29)	3 (10%)
			Naproxen (n17)	3 (18%)
			Pregabalin (n24)	2 (8%)
			Tramadol (n21)	-
Increased pain (7)	8 (6%)	16 (5%)	Amitriptyline (n32)	3 (1%)
			Celecoxib (n66)	3 (5%)
			Clonidine (n26)	-
			Gabapentin (n22)	-
			Melatonin (n29)	4 (14%)
			Naproxen (n17)	3 (18%)
			Pregabalin (n24)	-
			Tramadol (n21)	1 (5%)
Others (8)	15 (10%)	15 (5%)		

### 3.1 Patients reporting adverse effects (AEs)

One hundred fifteen patients (81%) reported AEs with a mean 2.0 ± 3.5 AEs per patient. Table 1 summarizes the incidence of AEs per patient, prescriptions and medications. Across 142 patients and 291 prescriptions, adverse effects were most commonly gastrointestinal and attention-related, affecting roughly two-thirds of patients (GI: 62%; attention: 61%) and appearing across about three-quarters of prescriptions (GI: 73%; attention: 73%). Gastrointestinal AEs included abdominal pain or fullness, constipation or diarrhea, decreased or increased appetite, nausea or vomiting, weight loss or gain, unpleasant taste, and dry mouth. AEs affecting attention included decreased concentration, somnolence, dizziness, drowsiness, memory impairment, fatigue, sedation, blurred vision, and involuntary eye movements.

Less frequent but present AEs included sleep disturbances, skin reactions, mood changes, and weakness/movements, each occurring in a smaller but notable share of patients and prescriptions. AEs related to uncoordinated movements and weakness included tremor, decreased coordination of fine movements, and vertigo. More frequent sleep disturbances AEs included nightmares and insomnia. Skin reactions AEs included itching, bruising, rash, sweating, swelling, and cold sensation.

Almost one in five patients reported mood changes (irritability, depression, anxiety, hallucinations, hostility), and one in ten reported increased pain (generalized pain, joint pain, muscular pain/spasms), each occurring in a smaller but notable share of patients and prescriptions.

No serious adverse effects or deaths were attributed to pharmacological treatment during this quality improvement analysis.

### 3.2 AEs and multiple medications

Ninety-three patients (65%) reported two or more types of AEs per medication. The proportion of patients reporting AEs increased with the number of concomitant medications. Among those on one drug, 50 of 69 patients (72%) reported AEs; among those on two drugs, 25 of 31 (81%) reported AEs; and among those on three or more drugs, 20 of 21 (95%) reported AEs.

### 3.3 Perceived benefits

**Figure 2:**
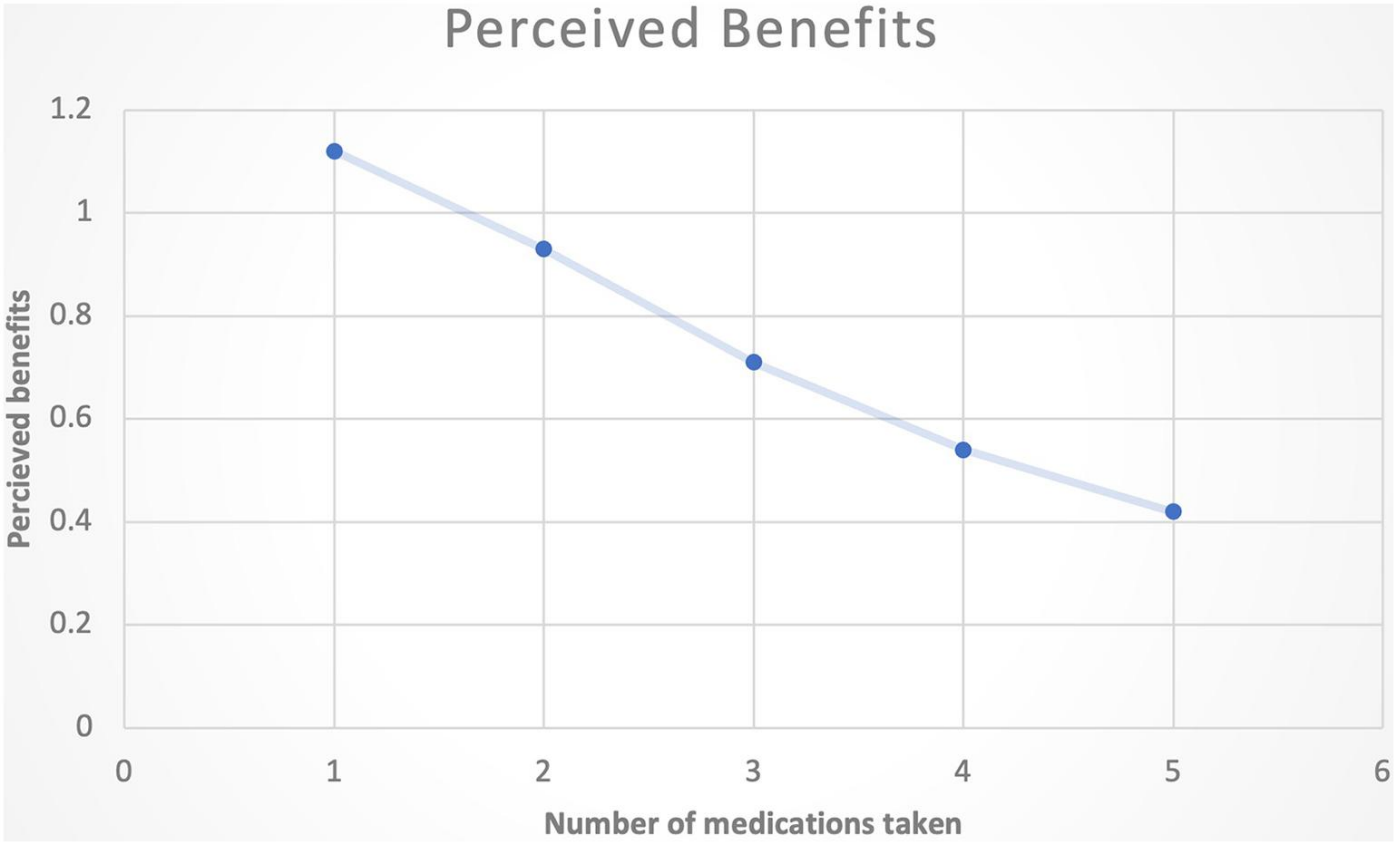
Graph depicting the diminishing perceived benefits with increased number of medications taken.

One hundred and nine patients (77%) perceived benefits in at least one domain (pain, sleep, physical function or mood) within four weeks of starting a new treatment. Table 2 describe the perceived benefits in relation with the number of medications received. Two thirds of patients (64%) referred analgesic benefit (reduction on intensity, frequency, or duration). The proportion of patients referring analgesic benefits remained relatively high across the number received medication. Sleep benefits were identified by 47% of patients who reported better sleep quality and less sleep disruptions due to pain. Physical functional improvements were reported 27% of the patients and mood improvements were identified by (22%) of patients. Overall, the mean number of benefited per patient increased with polypharmacy, suggesting broader patient-perceived benefit in this quality improvement study. However, the number of perceived benefits per prescription decreased with the number of medications prescribed (Figure 2).

**Table 2:** Patients reporting benefits within four weeks after starting a medication.

Medications per patient	Patient reporting any benefit	Benefits per patient	Patient reporting analgesic benefit	Patient reporting improvement in physical function	Patient reporting improvements in mood	Patients reporting improvements in sleep
1 (n69)	44 (64%)	1,8	36 (81%)	13 (30%)	7 (16%)	23 (52%)
2 (n31)	26 (83%)	2,2	22 (86%)	12 (46%)	8 (30%)	15 (58%)
3 (n42)	39 (90%)	2,4	32 (76%)	14 (33%)	16 (38%)	31 (73%)

## 4 Discussion

This quality-improvement study in a pediatric interdisciplinary chronic-pain program evaluated the incidence of AEs and patient-perceived benefits one month after initiating new pharmacological interventions within a real-world context. We collected patient-reported AEs and domain-specific benefits to inform safety surveillance and daily clinical practice. Our findings suggest that AEs are common and span several domains, with a pattern suggesting greater safety burdens as medication load increases. At the same time, patients frequently reported benefits across multiple domains, and these benefits tended to emerge as regimens became more complex, while the overall impact per prescription appeared to diminish with heavier polypharmacy. Importantly, no serious harm was attributed to treatment in these analyses.

### 4.1 Adverse effects

One of the understudied aspects of chronic non-oncological pain in children is the incidence of AEs. The incidence of AEs in pharmacological studies is surprisingly low compared to what we observed in our study (7–9, 19). Our findings suggest the presence of common AEs in a real-world setting. However, they should be interpreted as descriptive signals rather than definitive evidence of drug-specific harm. Formal causality assessment across all adverse effects was not feasible in our quality improvement study. AEs were recorded as routine-care events; consequently, causal inference and severity grading are limited. Our results should be interpreted as descriptive signals rather than drug-specific effects. Addressing causality would require ADR-focused protocols and validated outcomes within a different methodology (10, 20, 21).

The observed AE rates may reflect both true pharmacologic risk and the influence of structured data collection that can amplify safety signals through nocebo-like effects. In counseling, we highlight the most frequent AEs to reduce distress while guiding monitoring for rarer events. We provide clear steps on what to monitor, when to contact a clinician, and how to seek urgent care for red flags. We stress that benefits accompany the risks and that monitoring plans are shared. Risk communication was tailored to each patient and family. To reduce nocebo effects, we frame common AEs with approximate frequencies and acknowledge rare AEs without overstating their likelihood. Additionally, we use plain language and actionable guidance on symptoms, contacts, and care pathways. However, we cannot exclude a potential nocebo effect may be driven by the methodology used in this quality improvement study (12, 22).

Interpreting AE frequencies by medication requires caution. Many patients used multiple drugs and per-drug samples were small, so these figures do not estimate drug-specific risk. This quality improvement study did not quantify incidence attributable to individual meds or disentangle interactions. Instead, results show a wide range of AEs across medications in a polypharmacy context, highlighting broad safety considerations in multidisciplinary pediatric chronic-pain care. Interpreting per-medication risk has ethical and clinical implications for communicating risks and benefits to families. Our findings should be signals for safety surveillance and shared decision-making, not as causal evidence for any single medication.

The findings in this QI analysis suggest the potential hazards of polypharmacy and the risks of pharmacological interactions in children with chronic conditions. The observed increase in AEs with a greater number of concomitant medications is consistent with prior real-world data and can be driven by multiple, non-mutually exclusive mechanisms. (Kleykamp BA 2022). First, exposure burden increases as more drugs are prescribed, raising the chance that any individual drug will contribute an AE. Second, potential pharmacodynamic and pharmacokinetic interactions may amplify or reveal AEs not evident when drugs are used in isolation (23). Our study cannot disentangle these competing explanations because we did not perform formal causality or interaction analyses across medications. Consequently, the magnitude of interaction effects vs. exposure effects remains unclear.

### 4.2 Perceived benefits

There is currently minimal data available to support the benefits of pharmacological treatments in the management of chronic pain in this population (9, 10). Additionally, some studies have shown no perceived analgesic effects when comparing these medications to placebo (7–10). In our quality improvement study patients reported an increased number of perceived benefits with polypharmacy. This may be attributed to the complexity of pain, which is not purely a nociceptive experience, but rather a whole-person experience, as pain has been shown to be affected by emotions, mood, physical activity, as well as by the placebo effect (16, 17, 24). Perceived benefit tends to rise with more medications probably because different drugs address distinct domains such as pain, sleep, function, and mood, which can co-occur. Addressing multiple domains can yield net improvement even if each medication offers only modest benefit (7–10).

The decrease in benefit per prescription can be explained by several factors: greater adverse-event burden with more drugs; dosing and adherence challenges; fragmented or duplicated targets; limited time to evaluate many drugs; and misalignment between clinician targets and patient goals. Additional contributors include caregiver burden, communications among prescribers, and evolving expectations as treatment changes (12). These patterns underscore the need for integrated, patient-centered medication review and streamlined regimens to maximize benefit and minimize burden.

Perceived benefits were captured using simple, unanchored patient-reported question: “does this medication help you?” This emphasizes the patients' perspective over intensity or numerical scales. While easy to administer and interpret, this approach does not yield a quantified effect size, and responses may reflect expectations or reporting biases (12). Future work could incorporate validated patient reported outcome measures (e.g., global impression of change, pain interference) and qualitative interviews to better characterize drivers of perceived change.

### 4.3 Clinical evaluation of adverse effects and perceived benefits

The Lancet Child & Adolescent Health Commission set four goals to improve life for young people with chronic pain: make pain matter, understood, visible, and better (24). The Core-OPPP Workgroup recently recommended routine self-assessment tools to collect adverse and unintended events in pediatric pain trials supplemented by symptom checklists (25). Our QI analysis responds to the call for research on efficacy and safety made by the previous consensus studies.

Assessing risk and benefit as a combined metric at an individual level can reveal clinical responses that may not be obvious when assessing risk and benefit as separate outcomes. Kleykamp et al. compiled several recommendations to improve design and interpretation of chronic pain clinical trials, such as the standardization of terminology as well as steps to ensure proper risk-benefit assessment, which include the need for specificity, identifying outcomes and assessments, evaluating endpoints and analysis, interpreting the data, and communicating results of the analysis (20).

Risks and benefits could be systematically assessed at an individual level to enhance the clinical meaningfulness. Harm reduction policies in clinical practice advocate for a careful risk and benefit discussion and evaluation at the time of medication prescription and during follow-up appointments (10). Furthermore, quality improvement uses standardized protocols and PDSA cycles to test changes in real time. QI focuses on how a program works in daily practice, not just ideal trials. Our aim was to monitor safety and real-world value using routine data with minimal burden. This model of analysis fits within current workflows and regulatory needs. It also involves clinicians, patients, and families in data collection, boosting acceptability and the likelihood of sustained safety monitoring and benefits.

Although inferential statistics were not required in our study, exploratory analyses could strengthen the work. The sample's heterogeneity—multiple diagnoses, broad age range, and varied regimens—and the modest size limit meaningful stratified analyses and increase the risk of overinterpretation. Despite limited power for subgroups, the study provides a real-world view of safety and patient-perceived benefit, yielding actionable signals for program optimization and safety surveillance. AEs were documented as routine-care events without formal causality adjudication. While formal causality is central to pharmacovigilance and trials, applying such analyses in heterogeneous, interdisciplinary pediatric settings remains challenging. Our results address practical questions for clinicians and program directors, such as how common AEs are and whether higher medication load is associated with greater burden, and lay the groundwork for future prospective studies with predefined strata and validated outcomes.

### 4.4 Clinical framework

Clinicians can view our findings as a cue for proactive safety surveillance and shared decision-making in a setting of interdisciplinary pediatric chronic pain. When adding or reducing medications, use a brief, structured safety check during routine visits or short follow-ups to capture AEs and domain-specific benefits from patients and families. Document which domains show benefit (pain, sleep, function, mood) and which AEs predominate. Then use this information to guide tapering or simplification of the treatment. Communicate clearly about attribution uncertainty in a polypharmacy context, set realistic expectations for short-term vs. longer-term tolerability, and involve families in monitoring frequency, target symptoms, and re-evaluation milestones.

Adopt a routine practical approach for AE monitoring at medication changes and at regular follow-ups. Record AEs and domain-specific benefits from both patients and caregivers, along with timing relative to therapy changes. Include concomitant medications and intercurrent illnesses to contextualize safety data. Capture domain-specific benefits as independent indicators alongside the global impression of change to support patient-centered decisions about continuation, tapering, or simplification. We acknowledge that broader benefits may arise from addressing multiple domains, not single-drug effects, and tailor decisions accordingly (Table 3).

**Table 3:** Practical suggestions for the evaluation of adverse effects in clinical practice.

Clinical framework for assessment of adverse effects
Implement routine AE monitoring using standardized AE collection tools at baseline, medication changes and at follow-up (4 weeks, then every 6–8 weeks)
Collect domain-specific AEs (Gastrointestinal, attention/cognition, sleep, mood, etc.) and patient-perceived benefits to adjust regimens quickly and safely.
Promote integrated shared decision-making with patients and families reviewing patterns of AEs and benefits discussing short- and long-term outcomes including validated tools like the Patient Global Impression of Change
Use findings to guide deprescribing or regimen simplification when safety burdens outweigh benefits.
Be especially cautious with higher medication loads; tailor targets and pacing of therapy.

For implementation and governance, we recommend integrating a simple, repeatable workflow: an AE checklist, a domain-specific benefit form, a brief causality note, and a decision log with agreed actions. Use follow-up dashboards to track AEs, benefits, and treatment intensity, and discuss results with families in plain language, noting attribution uncertainties and follow-up plans.

This framework supports continuous improvement: collect data, interpret within attribution limits, adjust practice, and re-evaluate. It also highlights opportunities for pharmacovigilance analyses, causal modeling of drug interactions, and patient-centered outcomes using validated measures. When feasible, supplement routine data with severity scales for AEs and validated PROs to strengthen risk-benefit assessments and cross-study comparability.

### 4.5 Limitations

We want to address several limitations of our study. This QI project was conducted in a single multidisciplinary pediatric chronic-pain program. The cohort was small and followed for a short period, limiting the ability to draw causal inferences or generalize to other settings or populations. AEs were captured during routine care without formal severity grading or causality assessment, making attribution to specific medications difficult. The study addressed a narrow set of chronic pain conditions, though these are representative of what is seen in university hospital settings.

Additionally, AE reporting relied on unstandardized methods and could be influenced by reporting bias. Per-prescription AEs illustrate exposure burden but do not determine drug-specific risk or interactions. Forms completed by both patients and parents may introduce additional confounding factors. The use of a structured reporting prompt could heighten awareness of AEs, potentially inflating reporting (nocebo-like effects).

Finally, there was no control group or temporal analysis linking specific outcomes (for example, reductions in pain) directly to adverse effects across medications. Heterogeneity in diagnoses, developmental stages, and treatment regimens further complicates attribution and subgroup interpretation. Self- and caregiver-reported outcomes may be affected by recall bias. Taken together, findings should be viewed as descriptive signals intended to inform safety surveillance and patient-centered care in routine practice.

### 4.6 Strengths of the study

Across these lines, the goal remains to balance methodological rigor with real-world relevance. Combined approaches will help translate findings into practice, guiding safer, more personalized care for children with chronic pain. Despite its limitations, the information provided by this study could be considered as a step forward to address the literature gap regarding the adverse effects of pharmacological interventions in children and adolescents with chronic pain conditions. This quality-improvement study does not answer all questions about risk and benefit of pharmacological treatment. However, it provides descriptive, real-world signals about adverse effects and patient-perceived benefits from a real-world setting.

We do not claim efficacy or causal links for any regimen; causality requires formal pharmacovigilance and controls. The findings support proactive safety monitoring, transparent shared decision-making, and thoughtful deprescribing when risks outweigh benefits. We also show the potential value of tracking domain-specific outcomes alongside AEs to guide personalized care in routine interdisciplinary practice. The study highlights the need for transparency within the clinical team and with patients and families to build trust, clarify uncertainties, and support patient-centered decisions.

### 4.7 Future directions

Prospective, multicenter pharmacovigilance designs are needed to separate true pharmacologic risk from exposure burden. These studies should include explicit causality and drug–drug interaction analyses, along with longer follow-up to distinguish transient from durable AEs. Standardized AEs severity grading and validated patient-reported outcomes covering domain-specific and global goals will enhance precision and cross-study comparability. Emphasize person-centered phenotyping that goes beyond diagnosis to consider age, sex/gender, development, pain phenotype, and psychosocial context shaping safety signals and perceived benefit.

Research should examine how shared decision-making influences reporting and outcomes. This includes evaluating prompting methods for AE capture and potential nocebo effects to refine data collection and interpretation. Consider naturalistic designs that use controls such as medication-naïve patients or stepped-wedge cohorts within multidisciplinary programs may help to discern additive vs. synergistic benefits and to map optimal prescribing trajectories.

Emerging work should explore artificial intelligence (AI) and precision medicine in pediatric chronic pain. AI can analyze data, improve diagnostics, and predict treatment responses, including medication reactions and regimen adjustments. Real-world applications remain limited, so efforts should focus on translating AI insights into practical, safe clinical workflows (26).

Clinical trials have typically evaluated benefits and risks separately (21, 27–30). Methods that analyze both concurrently can use qualitative and quantitative approaches for group-level evaluation. Individual benefit–risk assessments enable cross-treatment comparisons and can be tailored to trial needs or to subgroups (e.g., age, pain type). Integrated assessments require detailed outcomes and both short- and long-term endpoints (20). Composite outcomes in analgesic pediatric trials are rare and require validation in pediatric populations (10).

## 5 Conclusion

The result of this quality-improvement study of pediatric chronic non-cancer pain treated with pharmacologic regimens suggested that adverse effects were common during the first four weeks, especially in polypharmacy contexts. Our findings describe the incidence of AEs and patient-perceived benefits in a real-world, multi-medication setting while not establishing causality between specific medications or quantifying drug–drug interactions or efficacy. Importantly, the results should not be interpreted as evidence of inefficacy or harm attributable to any single drug class. The study highlights safety surveillance challenges and emphasizes the value of incorporating patient and family perspectives into routine prescribing decisions. These observations provide a foundation for future prospective work that explicitly assesses causality and interactions, examines longer-term tolerability and benefit using standardized severity grading and validated patient-reported outcomes, and investigates whether safety signals differ across drug classes in controlled designs.

## Data Availability

The original contributions presented in the study are included in the article/Supplementary Material, further inquiries can be directed to the corresponding author.
